# Bio-Psycho-Social Approach to Reproductive Mental Health and Reproductive Decisions

**DOI:** 10.3390/bs13010075

**Published:** 2023-01-16

**Authors:** Eleonora Bielawska-Batorowicz

**Affiliations:** Institute of Psychology, Faculty of Educational Sciences, University of Łódź, al. Rodziny Scheiblerów 2, 90-128 Łódź, Poland; eleonora.batorowicz@now.uni.lodz.pl

The reproductive period in the human life cycle covers a great part of a person’s existence and is associated with many significant life changes. Although based on biological processes human reproduction has its social and psychological consequences and is embedded in a variety of cultural standards and expectations. While becoming a parent introduces new tasks and duties, it is also usually associated with joy and happiness, which is expected in most societies. However, for some people, it may be a source of existential and emotional crises or even serious mental health problems. The term reproductive mental health, used in the title of this editorial and in the title of the Special Issue of *Behavioral Sciences*, covers the broadly understood well-being of both women and men strictly related to their reproductive decisions, activities and their outcomes. It might be applied not only to factors influencing emotional disorders, but also to factors affecting individual well-being and family functioning. Such a broad concept is congruent with the bio-psycho-social perspective on both general health and mental health. The bio-psycho-social approach is particularly appropriate for analyses of human reproduction as it allows us to grasp different factors and mechanisms related to reproductive decisions and events, and puts them in a wide cultural context. Such an approach—as stated in earlier texts, e.g., [1]—should be applied not only in research but also in clinical work with clients who face problems while fulfilling their reproductive potential.

The issues traditionally associated with reproductive mental health include perinatal depression and anxiety, mostly experienced by women. It is worth reminding the readers that the list could be much longer and could include changes in mood during other periods of hormonal breakthroughs (e.g., menopause), as well as emotions and cognitions related to reproductive failure, such as miscarriage or infertility. Furthermore, men’s well-being might be affected by reproductive events too, with the couvade syndrome being one example [2] and men’s experience of childlessness being another example [3]. Nevertheless, the research on well-being in the perinatal period is concentrated more often on female than on male participants, and the papers presented in this Special Issue are not the exception. The increased involvement of fathers in all aspects of childcare—from their presence in delivery rooms to paternity leaves—reflects the change of parental roles and partnership in many societies. It is an important aspect of social context of human parenting and one can observe it as an ongoing process. That should increase the interest in men as research participants as without the focus on men it would not be possible to analyze all aspects of human reproduction. 

The recent COVID-19 pandemic provided new arguments for adopting a broader perspective when analyzing human reproduction and for the applicability of the bio-psycho-social approach. As a global health hazard, the pandemic influenced all areas of individual and social life. It also affected reproductive mental health and decisions. As presented in many studies, COVID-19 increased the incidence of anxiety, other psychopathological symptoms and risky behaviors, e.g., [4,5,6]. It also affected people of reproductive age as it increased the risk of perinatal depressive symptoms [7] and brought forward concerns related to effects of infection during the course of pregnancy and on the fetus [8]. Thus, it increased the burden and stress experienced by parents in the perinatal period. Our collection includes papers that also address pandemic issues. The results of the study by Studniczek and Kossakowska [9] indicate that although pandemic-related stress poses a risk for an increase in perinatal depressive symptoms, a high level of resilience is the most significant protective factor against such symptoms. Thus, another example of the positive role of psychological personal resources was presented—the research evidence indicated that such resources (i.e., resiliency) might improve perinatal maternal mental health by diminishing the level of depressive symptoms. Concerns related to COVID-19 effects on pregnancy might postpone decisions to conceive as indicated in the study by Albeitawi et al. [10], also included in this Special Issue. Such decision might be relatively easier to make in the fertile population as the study has indicated. Those who were engaged in the process of infertility treatment were much more distressed and despaired when the pandemic forced them to change their treatment schedule or postpone it altogether, which was indicated by other studies [11,12] as well. Thus, the pandemic as a global universal factor might affect individual experiences and private matters, such as mood during pregnancy and the decision to start a family. Such decisions can also be influenced by another global factor—climate change. The possible effects that perceiving such changes can have on reproductive decisions were examined in another paper in this Special Issue [13]. The findings indicate that positive reproductive intentions decrease with climate concerns, namely, with concerns about the effects of climate change on the health of individuals.

The application of the bio-psycho-social approach allows us to consider different aspects of human reproduction—from biological mechanisms that enable or preclude conception to individual psychological reactions to any reproductive event to the social and cultural context of any reproductive decision. Furthermore, it allows us to look at the relations between biological, psychological and social factors and to consider these relations as systemic, that is, reciprocal and mutually effective. Therefore, it is worth considering that the complexity and variety of factors should be taken into account every time reproductive mental health or reproductive decisions are analyzed. Some factors might be relatively more important in certain circumstances or for particular people and couples, but it does not mean that other factors and their complexity could be ignored either in research or in clinical work. The recent pandemic and observed climate change add a much more global perspective to the bio-psycho-social approach. It is not only one’s society that influences a person’s life. There are many other societies, with their lifestyles and technologies, that affect both their own members and those living on other continents as well.
